# Early Diagnosis and Treatment of Purine Nucleoside Phosphorylase (PNP) Deficiency through TREC-Based Newborn Screening

**DOI:** 10.3390/ijns7040062

**Published:** 2021-09-29

**Authors:** Andrea Martín-Nalda, Jacques G. Rivière, Mireia Català-Besa, Marina García-Prat, Alba Parra-Martínez, Mónica Martínez-Gallo, Roger Colobran, Ana Argudo-Ramírez, Jose Luis Marín-Soria, Judit García-Villoria, Laura Alonso, Jose Antonio Arranz-Amo, Giancarlo la Marca, Pere Soler-Palacín

**Affiliations:** 1 Pediatric Infectious Diseases and Immunodeficiencies Unit, Hospital Universitari Vall d’Hebron, Vall d’Hebron Research Institute (VHIR), Universitat Autònoma de Barcelona, 08035 Barcelona, Spain; jriviere@vhebron.net (J.G.R.); mirecatala@gmail.com (M.C.-B.); marinagarcia@upiip.com (M.G.-P.); albaparra@upiip.com (A.P.-M.); psoler@vhebron.net (P.S.-P.); 2 Immunology Division, Hospital Universitari Vall d’Hebron, Institut de Recerca Vall d’Hebron, Universitat Autònoma de Barcelona, 08035 Barcelona, Spain; mmartinez@vhebron.net (M.M.-G.); rogercol@yahoo.com (R.C.); 3 Jeffrey Modell Diagnostic and Research Center for Primary Immunodeficiencies, 08035 Barcelona, Spain; 4 Diagnostic Immunology Group, Vall d’Hebron Institut de Recerca (VHIR), Hospital Universitari Vall d’Hebron, Vall d’Hebron Barcelona Hospital Campus, Passeig Vall d’Hebron 119-129, 08035 Barcelona, Spain; 5 Departament de Biologia Cel·lular, Fisiologia i Immunologia, Universitat Autònoma de Barcelona, 08193 Bellaterra, Spain; 6 Genetics Department, Hospital Universitari Vall d’Hebron, Vall d’Hebron Barcelona Hospital Campus, Passeig Vall d’Hebron 119-129, 08035 Barcelona, Spain; 7 Newborn Screening Laboratory, Inborn Errors of Metabolism Division, Biochemistry and Molecular Genetics Department, Hospital Clínic de Barcelona, 08036 Barcelona, Spain; ARGUDO@clinic.cat (A.A.-R.); JLMARIN@clinic.cat (J.L.M.-S.); 8 Inborn Errors of Metabolism Division, Biochemistry and Molecular Genetics Department, Hospital Clínic de Barcelona, IDIBAPS, CIBERER, 08036 Barcelona, Spain; JUGARCIA@clinic.cat; 9 Pediatric Oncology and Hematology Department, Hospital Universitari Vall d’Hebron, Vall d’Hebron Research Institute (VHIR), Universitat Autònoma de Barcelona, 08035 Barcelona, Spain; l.alonso@vhebron.net; 10 Metabolic Diseases Laboratory, Hospital Universitari Vall d’Hebron, Vall d’Hebron Research Institute (VHIR), Universitat Autònoma de Barcelona, 08035 Barcelona, Spain; jaarranz@vhebron.net; 11 Department of Experimental and Clinical Biomedical Sciences, University of Florence and Newborn Screening, Clinical Chemistry and Pharmacology Lab, Meyer Children’s University Hospital, 50139 Florence, Italy; giancarlo.lamarca@meyer.it

**Keywords:** purine nucleoside phosphorylase deficiency, severe combined immunodeficiency, newborn screening, T-cell receptor excision circle

## Abstract

Purine nucleoside phosphorylase (PNP) deficiency is a rare inherited disorder, resulting in severe combined immunodeficiency. To date, PNP deficiency has been detected in newborn screening only through the use of liquid chromatography tandem mass spectrometry. We report the first case in which PNP deficiency was detected by TREC analysis.

## 1. Background

Severe combined immunodeficiency (SCID) is the most severe T-cell immunodeficiency, exposing children to immune dysregulation and recurrent opportunistic infections. SCID affects an estimated 1/50,000 to 1/100,000 births and is usually fatal in the first year of life unless promptly recognized and appropriately treated [1,2]. SCID can be reliably detected using newborn screening with dried blood spot (DBS) samples using T-cell receptor excision circle (TREC) analysis [1]. Among the accumulated experience of newborn screening (NBS) for several metabolic and other inherited diseases in Europe and worldwide [3], NBS for SCID was first implemented in Wisconsin in 2008 and was subsequently adopted in several geographic areas, including Catalonia (Spain) in 2017 [1,2,4]. Three SCID cases were diagnosed in Catalonia between 2017 and May 2020, yielding an approximate incidence of 1/65,000 births, a rate in accordance with the findings in previous reports [2].

Purine nucleoside phosphorylase (PNP) deficiency is a rare combined autosomal recessive primary immunodeficiency with associated non-immune features that accounts for approximately 4% of all SCID cases. It is characterized by progressive T-cell immunodeficiency and variable abnormalities of humoral immunity, with autoimmunity, malignancy, and neurological impairment [5,6]. PNP catalyzes the reversible phosphorolysis of inosine (Ino), guanosine (Guo), deoxyinosine (dIno), and deoxyguanosine (dGuo) to their respective purine bases and pentose-1-phosphates. The PNP substrates undergo alternative metabolism to toxic metabolites. The deoxyguanosine triphosphate (dGTP) derived from dGuo leads to profound impairment of lymphocyte maturation and progressive functional T-cell defects. Autoimmunity and uncontrolled cell proliferation are also common. Nonimmune manifestations mainly consist of neurological abnormalities, which affect more than 50% of patients. These abnormalities can precede infections or autoimmunity, in contrast to what occurs in other inherited immune defects [6,7]. Laboratory findings include lymphopenia and markedly reduced T-cell proliferation; however, these parameters may be normal in early life and gradually decrease because of the metabolites’ toxic effect [6]. Humoral immunity may be normal, but some patients show reduced B-cell counts with absent isohemagglutinins. A hallmark feature for the diagnosis of the disease is the accumulation of the substrate metabolites (dIno, Ino, dGuo, and Guo) in plasma and/or urine. The increases in these metabolites can also be detected in dried blood spot testing by tandem mass spectrometry (TMS) used for newborn screening [8]. In addition, most patients show low serum and urinary uric acid levels, as a result of impaired purine catabolism [6,9,10]. However, the diagnosis of PNP deficiency is established by markedly reduced (<5%) or absent PNP activity in erythrocyte lysates. Detection of a defect in the *PNP* gene helps to confirm the diagnosis and enables genetic counselling [6].

## 2. Case Presentation

Here, we describe the case of a 9-day-old (40-week pregnancy) asymptomatic single son of consanguineous Moroccan parents, referred to our unit because of positive newborn screening findings at 48 h of life (TREC count, 4 copies/μL—mean of 3 punches; cut-off <20 copies/μL). TREC quantification in DBS (1.5-mm diameter spot) was performed according to the Enlite Neonatal TREC kit instructions (PerkinElmer, Turku, Finland) [4]. Laboratory tests at 9 days of life showed mild leukopenia (4.02 × 10^9^/L), with lymphopenia (0.9 × 10^9^/L) affecting the absolute number of all lymphocyte subpopulations. The percentage of CD4+ T cells was normal, whereas the CD8+ T cell percentage was below the normal range for the age, B cells were present at 4%, and natural killer cells were increased at 45%. There was no evidence of basal T lymphocyte activation. The initial proliferative response to phytohemagglutinin (PHA) performed at 9 days of life was slightly decreased (45% below the control value), whereas a subsequent determination at 1 month of life was within normal values (Table 1).

Genetic analysis was performed by next-generation sequencing, using a panel of 323 PID-causing genes with a custom-designed targeted enrichment approach (SeqCap EZ ChoiceTM, NimbleGen, Roche, Basel, Switzerland). A novel, homozygous, and likely pathogenic missense variant was detected in exon 5 of the *PNP* gene, c.602A>G (p.Glu201Gly), and both parents were found to be heterozygous carriers. This variant shows a very low frequency in the Gnomad database (only 1 heterozygous allele among more than 250,000 tested; MAF = 0.000004). The Glu201 residue is highly conserved across species, and all the main computational predictors (e.g., SIFT, PolyPhen2, CADD) consider this variant to be damaging. Several other variants near Glu201 have been reported to cause PNP deficiency (e.g., p.Ser199Arg, p.Pro198Leu) [11].

As was expected, metabolic studies when using liquid chromatography showed increased Guo, Ino, dGuo, and dIno urine levels and low plasma uric acid (2.2 mg/dL; normal range: 3.5–7.2) (Table 2). PNP enzyme activity was low in tests on DBS samples taken at 48 h of life: 11 nmol/mg·h (reference range 823–2387 nmol/mg·h) (Laboratory Genetic Metabolic Diseases [F0-132], Amsterdam UMC; Location: AMC Meibergdreef 9 1105 AZ, Amsterdam, The Netherlands), supporting the diagnosis. PNP enzyme activity measured in plasma samples from the patient’s father and mother was low (659 and 542 nmol/mg·h, respectively) and was consistent with their carrier status.

The infant was hospitalized and provided with protective isolation and antimicrobial prophylaxis (trimethoprim-sulfamethoxazole). At that time point (30 days of life), the neurologic examination was normal, showing no developmental delay, spasticity, or sensorineural deafness. No matched related or unrelated donor, or suitable unrelated cord blood unit was available. Hence, the patient received a haploidentical hematopoietic stem cell transplant (HSCT) from his father, chosen because of the mother’s comorbidities, with a reduced-intensity conditioning-based regimen consisting of antithymocyte globulin (2.5 mg/kg/day, total dose 10 mg/kg), on days −10 to −7, busulfan with level monitoring and dose adjustment to achieve an AUC of 65,000 ng/mL × h total dose on days −6 to −3, and fludarabine 45 mg/m^2^/day (total dose 180 mg/m^2^) on days −6 to −3. The stem cell apheresis product was G-CSF-mobilized PBSC, which underwent CD34 selection and CD3 dose adjustment to 1 × 10^5^/kg. The characteristics of the final product were as follows: TNC/kg 0.363 × 10^8^, CD34/kg 14.09 × 10^6^, CD3/kg 1 × 10^5^, and viability 44%. Graft-versus-host disease (GvHD) prophylaxis consisted of cyclosporine and mycophenolate.

The infant was discharged from the bone marrow transplant ward 44 days after transplantation with no infections or GvHD and 100% donor chimerism. However, 2 months after HSCT he was admitted with pancytopenia of unknown origin and mixed chimerism with 67% donor and received a CD34+ selected stem cell boost, which led to improvements in cell counts, while lymphopenia and lymphoid mixed chimerism persisted. For this reason, 6 months after HSCT, a donor lymphocyte infusion was administered. The latest follow-up was at 11 months of life. Up to the time of writing, the neurological examination has shown signs of mild spasticity, and lymphoid donor chimerism is 60%.

## 3. Discussion

TREC-based newborn screening using a quantitative PCR method in eluted DNA from routinely collected DBS samples has proven to reliably detect SCID and other T-cell disorders in asymptomatic infants. This capability enables an early diagnosis and prompts clinical management, which is related to a significant decrease in morbidity and mortality compared to the outcome in cases diagnosed when clinical features of recurrent infections and progressive neurologic symptoms have occurred [1,2,4]. However, to our knowledge, this is the first reported patient with PNP deficiency detected with this approach; while other cases have been reported using TMS [8]. While other studies performed retrospectively after the genetic diagnosis showed reduced TREC in DBS and in whole blood DNA extraction [14], this was not the case in the publication by Brodszki N et al. [15], showing than PNP deficiency should be suspected with compatible clinical features, even in NBS with TRECS that yielded normal results. Previous reports have cited TMS on DBS as an accurate approach for this purpose [1,2,14,16]. To confirm this point, in our case, a metabolite study from the original DBS was performed using TMS, which showed an increase in Guo, dGuo, and dIno, as has been previously described [8].

Currently, the only effective therapy for PNP deficiency is allogeneic HSCT [6,7,16]. HSCT restores lymphoid cells with PNP activity, particularly peripheral blood granulocytes and lymphocytes. It also allows metabolic detoxification of other organs before irreversible damage occurs [6,7]. In this sense, while some studies have suggested that neurological deficits often remain or progress despite early treatment [16,17], other reports have shown that HSCT at an early age may be beneficial to prevent further neurological injuries, and to stabilize (but not reverse) those already present [7,15].

To conclude, this case shows that PNP can be promptly detected in newborn screening by TREC-based PCR, at least in some patients. Early stem cell transplantation may restore purine nucleoside metabolism in non-neuronal cell populations and prevent secondary neuronal damage in these cases.

## Figures and Tables

**Table 1:** Characteristics of the patient’s lymphocyte subpopulations and lymphocyte proliferation.

Parameter	9 Days of Life	20 Days of Life	1 Month Old	Reference Values
Leucocytes, ×10^9^/L	4.02	4.21	3.99	5.00–20.00
Lymphocytes, % (×10^9^/L)	22.9 (0.9)	28 (1.2)	24.8 (1)	25.0–60.0 (3.4–7.6)
CD3+, % (×10^9^/L)	50.18 (0.46)	50.21 (0.50)	51.45 (0.51)	52–77 (1.85–5.96) *
CD3+ CD4+, % (×10^9^/L)	44.63 (0.41)	42.23 (0.42)	44.07 (0.44)	30.00–58.00 (1.14–3.8) *
CD3+ CD8+, % (×10^9^/L)	4.64 (0.04)	3.75 (0.04)	4.01 (0.04)	12.00–27.00 (0.54–1.97) *
CD19, % (×10^9^/L)	4.68 (0.04)	9.45 (0.09)	13.3 (0.13)	15–28 (0.64–1.96) *
CD56+ CD16+ CD3−, % (×10^9^/L)	45 (0.41)	37.97 (0.38)	28.79 (0.29)	3–24 (0.15–1.33) *
CD4/CD8 INDEX	9.62	11.26	10.99	1.3–6.30
CD3+ TCRαβ, %	91	N.A.	N.A.	83–97
CD3+ TCRγδ, %	6.8	N.A.	N.A.	2–15
CD4+ CD45RO, %	37	N.A.	N.A.	<15
CD8+ CD45RO, %	13	N.A.	N.A.	<15
CD4+ HLA-DR+, %	6.9	N.A.	N.A.	0–5
CD8+ HLA-DR+, %	13.4	N.A.	N.A.	0–5
CD4+ RTE, %	27.25	N.A.	N.A.	47–79 *
PMA + ionomycin, % of control	100 (Normal)	>100 (Normal)	N.A.	
PHA, % of control	45 (Low)	>100 (Normal)	>100 (Normal)	
PWM, % of control	>100 (Normal)	>100 (Normal)	N.A.	

Altered values are marked in bold. * Reference values for children from 0 to 2 years old [12] (Garcia-Prat et al., Cytom. B Clin. Cytom. 2019). N.A.: Not assessed

**Table 2:** Metabolic studies in urine (26 days of life).

Parameter	Patient (mmol/mol Creat)	Reference Values *
Guanosine	439.7	<1.5
Deoxyguanosine	284.2	<0.1
Inosine	510.0	<3.1
Deoxyinosine	228.1	<0.2

* Reference values for children. Adapted from [13] (van Kuilenburg ABP et al., 2008, in Blau, Duran, Gibson, eds.: Laboratory Guide to the Methods in Biochemical Genetics).
